# Investigating Single-Molecule Molecular Inversion Probes for Medium-Scale Targeted DNA Methylation Analysis

**DOI:** 10.3390/epigenomes9010008

**Published:** 2025-03-02

**Authors:** Roy B. Simons, Hieab H. H. Adams, Manfred Kayser, Athina Vidaki

**Affiliations:** 1 Department of Genetic Identification, Erasmus MC, University Medical Center Rotterdam, 3015 GD Rotterdam, The Netherlands; 2 Department of Clinical Genetics, Erasmus MC, University Medical Center Rotterdam, 3015 GD Rotterdam, The Netherlands

**Keywords:** age prediction, capture, DNA methylation, epigenetics, padlock probes, single-molecule molecular inversion probes, unique molecular identifiers

## Abstract

Background: Epigenetic biomarkers, particularly CpG methylation, are increasingly employed in clinical and forensic settings. However, we still lack a cost-effective, sensitive, medium-scale method for the analysis of hundreds to thousands of user-defined CpGs suitable for minute DNA input amounts (<10 ng). In this study, motivated by promising results in the genetics field, we investigated single-molecule molecular inversion probes (smMIPs) for simultaneous analysis of hundreds of CpGs by using an example set of 514 age-associated CpGs (Zhang model). Methods: First, we developed a novel smMIP design tool to suit bisulfite-converted DNA (Locksmith). Then, to optimize the capture process, we performed single-probe capture for ten selected, representative smMIPs. Based on this pilot, the full smMIP panel was tested under varying capture conditions, including hybridization and elongation temperature, smMIP and template DNA amounts, dNTP concentration and elongation time. Results: Overall, we found that the capture efficiency was highly probe-(and hence, sequence-) dependent, with a heterogeneous coverage distribution across CpGs higher than the 1000-fold range. Considering CpGs with at least 20X coverage, we yielded robust methylation detection with levels comparable to those obtained from the gold standard EPIC microarray analysis (Pearsons’s r: 0.96). Conclusions: The observed low specificity and uniformity indicate that smMIPs in their current form are not compatible with the lowered complexity of bisulfite-converted DNA.

## 1. Introduction

Epigenetics plays an increasingly important role in health and disease [1] as it indicates which parts of our genome are ‘active’ in different cells and cell types throughout life as a response to various environmental stimuli, i.e., aging, environmental effects, lifestyle or drug treatments. Notably, in cancer, cancer type-specific epigenetic signatures—particularly DNA methylation patterns [2], the most popular epigenetic modification—have been vastly identified, such as via The Cancer Genome Atlas Network [3]. Differential DNA methylation has also been shown to correlate with other diseases such as cardiovascular [4,5] and neurodegenerative diseases [6]. Generally, increased CpG methylation in promoter regions correlates with the suppression of gene expression [7]. Beyond biomedicine, DNA methylation has opened up research questions and applications in other, more specialized fields such as public health, epidemiology and even forensics [8]. Following the same path as genetics, DNA methylation is also highly personalized [9], a feature that makes it very powerful to investigate and apply in clinical and forensic settings. Particularly, differential DNA methylation levels can reveal and even predict a range of personal characteristics, such as chronological/biological age [10], tobacco smoking status [11,12], alcohol intake [13] and toxicant exposure [14]. For instance, predicting how ‘fast or slow’ an individual or tissue ages can be useful not only in assessing age-related disease risk(s) and in detecting disease-related abnormalities, but also in giving clues to the police to narrow down suspects in criminal investigations [15].

Currently, the most widely used method for genome-wide DNA methylation analysis are the Illumina methylation beadchip microarrays [16], which simultaneously type close to a million CpGs sites and, since 2011, have led the discovery of a myriad of methylation biomarkers across health and disease. For some clinical or forensic diagnostic purposes, however, DNA methylation microarrays are not practical for various reasons. First, such analysis requires a large amount of input DNA (200 ng) and the price per sample is quite costly [17]. Hence, microarrays are not suitable for low quality/quantity DNA samples often obtained in liquid biopsies in clinical setting as well as in crime scene traces in forensics. However, while not designed to handle such low inputs, methylation beadchip arrays have recently been shown to reach reproducible results when using as low as picogram DNA inputs [18]. The amount of missing data, however, as a consequence of their applications to low-input DNA, can form an issue, e.g., when applying existing, non-dynamic prediction models to such incomplete data. Moreover, given that they target a predefined set of methylation sites, microarrays offer no flexibility to the user for studying CpGs not covered by the commercially designed probe panel. Epigenome-wide microarrays as well as Reduced Representation Bisulfite Sequencing (RRBS), which enriches for CpG-dense regions across the genome [19], are great methods for large-scale analysis such as biomarker discovery [20,21]. However, when only hundreds or thousands of CpG targets are of interest, these methods are not suitable.

On the other hand, currently available targeted DNA methylation methods are suitable mainly for small-scale panels of up to about a dozen CpG markers, such as the qPCR-based method Methylight [22], single nucleotide primer extension-based SNaPshot [23] and standard primer-based multiplex bisulfite PCR [24]. To increase CpG coverage, global- and region-level methylation analysis has been successfully applied in clinical diagnostics through the detection of differentially methylated regions (DMRs) via genome-wide or targeted techniques, respectively [25,26,27]. This approach, however, lacks single-CpG resolution, which is often desired for clinical and forensic applications that utilize prediction models built with methylation microarray data [28]. Finally, another large-scale targeted method based on hybridization probe capture has been recently proposed [29]. In this method, target DNA is captured through hybridization on complementary DNA sequences attached to magnetic beads. However, this method can result in a significant loss of DNA and suffers from off-target reads [30], leading to low sensitivity and specificity.

Molecular inversion probes (MIPs), originally named as padlock probes [31], have been used for large-scale single nucleotide polymorphism (SNP) detection [32,33]. MIP capture as a targeted method for cost-effective SNP detection has been effectively applied for patient diagnosis and clinical outcome prediction [34] as well as for forensic purposes [35,36]. Although previous MIP production was a complex in-house production process that needed microarrays [37], oligonucleotide pools can now be directly ordered from commercial suppliers, making MIP capture more accessible to researchers. Additionally, including unique molecular identifiers (UMIs) counteracts the bias introduced by PCR, making it possible to distinguish single-capture events, creating the so-called single-molecule MIPs (smMIPs) [38]. So far, Diep et al. has applied an smMIP approach for the first epigenomic application using bisulfite-converted DNA [39]. The authors successfully typed >300,000 CpGs of interest, but their large-scale investigation still included large amounts of DNA; hence, the applicability of this approach to smaller-scale CpG panels and to compromised DNA still remains unknown.

**Figure 1:**
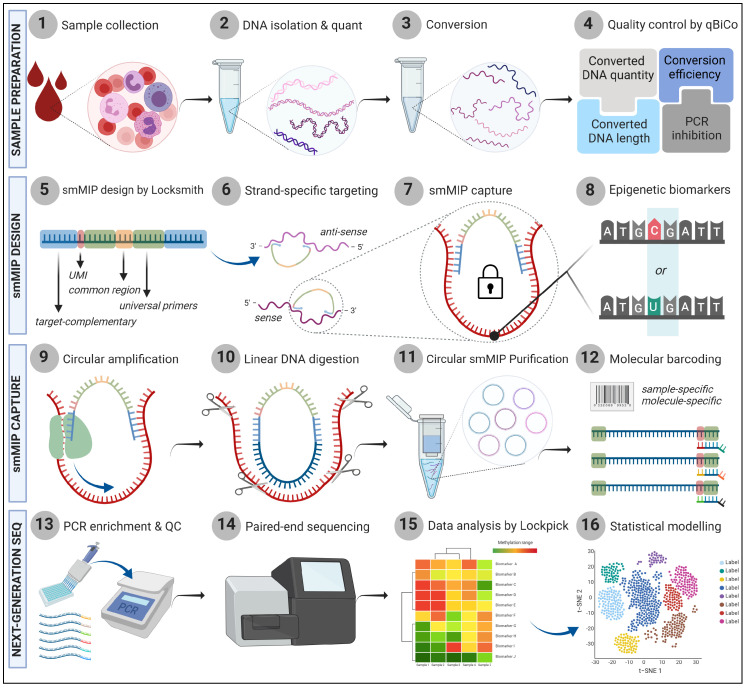
Schematic representation of targeted methylation profiling via smMIP sequencing. (**1**–**4**) Genomic DNA is extracted and bisulfite-converted to distinguish methylated from unmethylated cytosines; unmethylated cytosines are converted to thymines, resulting in the loss of complementarity between the two DNA strands. qBiCo is used to thoroughly evaluate the conversion process. (**5**–**8**) An smMIP panel is designed using Locksmith to target hundreds of CpGs in a strand-specific fashion. (**9**–**12**) smMIPs hybridize, circularize in parallel and are purified. Blue DNA strand: smMIP; Red DNA strand: bisulfite converted DNA fragment; Green ovals: DNA polymerase; scissors: Exonuclease. (**13**–**16**) Circular smMIPs are amplified using universal primers (one-step library preparation) and sequenced. Methylation data of target CpGs are analyzed with Lockpick, followed by downstream statistical analysis. Figure created with BioRender.com. smMIP: single-molecule Molecular Inversion Probe; UMI: Unique Molecular Identifier.

In this study, we aimed to investigate the use of smMIPs for targeted DNA methylation analysis of hundreds of CpGs simultaneously from low-input DNA amounts (Figure 1). By using 514 age-associated CpGs as an example [40], our goal was to evaluate smMIPs for medium-scale targeted DNA methylation analysis. This would be a tool that (a) is sensitive, suitable for low-input/quality DNA (10 ng); (b) offers medium-scale analysis for investigating hundreds or thousands of small regions; (c) is flexible for accelerating the implementation of diverse CpG panels across various fields; (d) offers high-resolution for strand- and CpG-specific analysis; (e) is highly robust, suitable for effective user-testing and future standardization; and, finally, (f) is cost-effective for affordable use in clinical and forensic settings.

## 2. Results

### 2.1. smMIP Panel Design Using Our Novel Locksmith Tool

**Figure 2:**
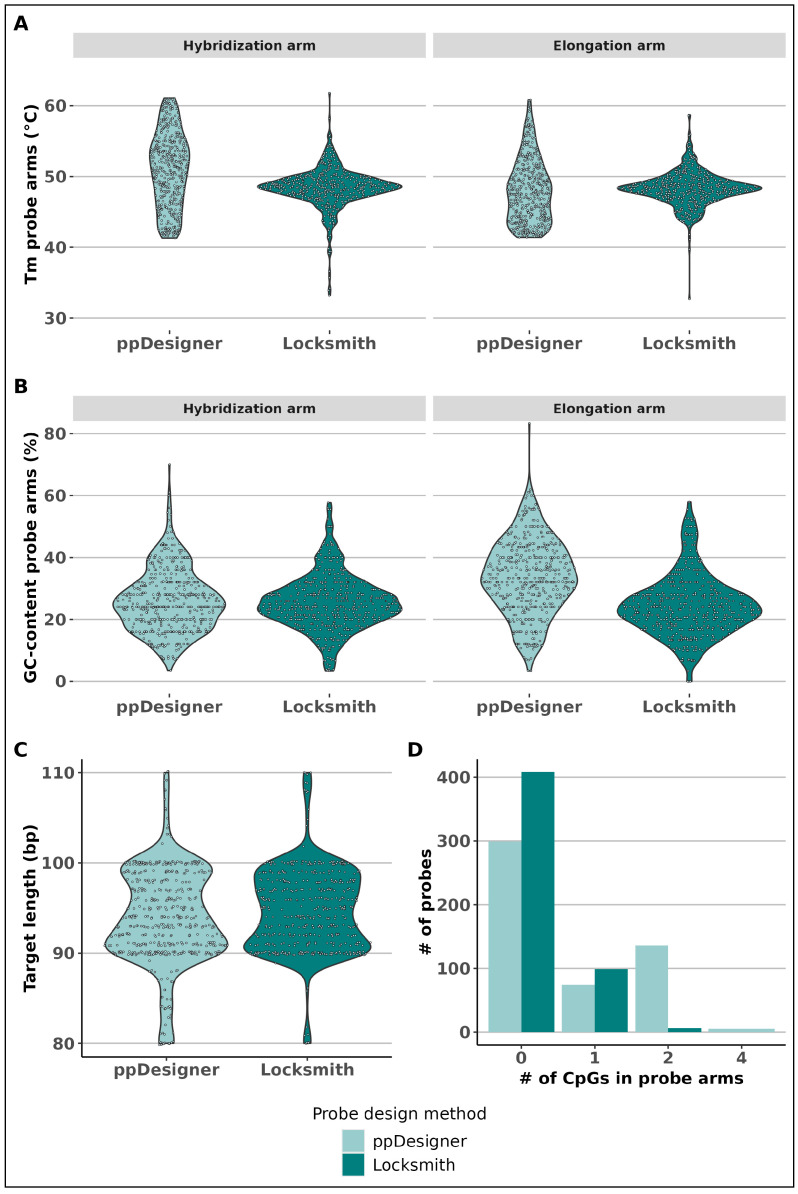
Comparison of designed probe parameters between ppDesigner and Locksmith for an 514-CpG panel. (**A**) Distribution of melting temperature for both hybridization and elongation probe arms. (**B**) Distribution of GC-content for both probe arms. (**C**) Distribution of sequence target length within captured products. (**D**) Number of probes containing different numbers of CG-dinucleotides in at least one of the arms.

To successfully apply the concept of smMIPs to targeted, medium-scale epigenomic analysis, we first aimed to design a novel smMIP design tool, which we specifically developed for bisulfite-converted DNA analysis. We designed Locksmith based on our previous experience, employing a similar probe design tool named ppDesigner [39]. ppDesigner focuses on the design of each single smMIP separately by scoring all possible smMIPs per target into a score combining target length, target GC-content, binding arm melting temperature, binding arm length, local single-stranded folding energy of the target and dinucleotides present at the extension and ligation sites during smMIP capture. The panel is then constructed by reporting the set of smMIPs with the highest efficiency. In contrast, the new Locksmith tool introduced and applied here takes into account smMIP monomer and dimer formation, thereby optimizing the full panel. In short, during probe design, Locksmith scores all designed smMIPs for each target CpG based on several parameters related to the smMIP arms while minimizing smMIP hairpins, monomers and dimers, which are expected to be commonly encountered due to the nature of bisulfite-converted DNA. These arm parameters include melting temperature, GC-content, arm length and the presence of CG-dinucleotides and frequently occurring SNPs. To construct a probe panel, Locksmith selects smMIPs by a random selection procedure weighted on the probe scores. Any conflicting smMIPs due to dimer-formation are re-scored, thereby decreasing the chance for selection. The panel is redesigned until no conflicts are present. We tested Locksmith by directly comparing its output with ppDesigner regarding our desired medium-scale panel, targeting 514 age-associated CpGs that are included in the recently published but well-received Zhang model [40]. Both panels were designed using an iterative design approach by iteratively loosening constraints to accommodate the design of probes in low-complexity or CpG-dense regions (Figure 2).

First, it was desired to minimize the difference between the melting temperatures of the various probes in order to accommodate similar probe capture efficiencies. When comparing the two panels side-by-side, we notice that both the hybridization and elongation arm melting temperatures of the designed probes were more unified for Locksmith with average Tms of 48.4 ± 2.8 °C and 48.3 ± 2.4 °C, compared to 51.0 ± 5.3 °C and 48.0 ± 4.4 °C for ppDesigner, respectively (Figure 2A). Secondly, GC-content also affects probe capture efficiency; therefore, uniformity between probes in the panel is desired. Here, Locksmith also showed a narrower GC-content distribution with 24.5 ± 9.8%, as opposed to 33.7 ± 11.9% for ppDesigner when we refer to the hybridization arm and 25.5 ± 8.8% as opposed to 26.0 ± 10.0% when we refer to the elongation arm, respectively (Figure 2B). Thirdly, minimization of the target length difference results in a more uniform length of the sequencing library, improving the possibility for stringent clean-up. Although the target length distributions were similar between both tools, ppDesigner resulted in more than double the number of probes, with a target length outside of the preferred 90–100 bp range with 66 probes, as opposed to the 28 probes by Locksmith (Figure 2C). Finally, minimization of the amount of CpGs in the probe arms is desired as the unknown methylation state of these CpGs at the hybridization locus can affect binding efficiency. This, however, was unavoidable for markers in CpG-dense regions; therefore, probes containing these arms were ordered with degenerate nucleotides. Locksmith probes had a total of 111 CpGs in both arms, whereas ppDesigner probes contained a total of 366 CpGs (Figure 2D), meaning that Locksmith relied less on degenerate nucleotides in the probe arms. Due to the implementation of penalizing probe arms that form secondary structures at the probe design stage, none of the Locksmith probes formed secondary structures; yet, the ppDesigner pool did result in the formation of six dimers above the melting temperature of 20 °C (Supplementary File S3). Interestingly, secondary structures do not seem to occur as often for smMIPs as for (multiplex) PCR primers. This is most likely due to the combination of (1) both arms of an smMIP targeting the same strand and (2) the depletion of guanines in the smMIP sequence due to the C-to-T conversion of the template DNA. This is not the case for multiplex PCR primers as smMIPs all target the converted DNA directly, whereas half of the primers in a multiplex PCR will target the complementary sequence of a converted DNA strand. The broad distributions of the probe parameters by ppDesigner inspired the implementation of probe scoring for Locksmith so that a more uniform probe panel could be designed. This comparison showed the possibility for improving the uniformity of the probe panel design and the probe design for CpG-dense regions.

### 2.2. Pilot Testing of a Small 10-CpG smMIP Panel

Prior to testing the capture efficiency of the full 514-CpG smMIP panel and to allow for some first insights to guide potential further tweaking of the Locksmith design approach, we aimed to test a small 10-smMIP panel in a pooled capture reaction. We chose ten smMIPS from the full panel design to represent probes of diverse characteristics including probes with melting temperatures at the highest variance and probe arms with degenerate nucleotides in CpG-dense regions. Directly following the 10-smMIP capture, ten separate PCR reactions were specifically designed and performed using synthetic DNA fragments to individually amplify and visualize the captured targets. Importantly, to ensure only captured targets were amplified without non-specific amplification of the template DNA, forward PCR primers were designed to anneal the target regions, while reverse PCR primers were designed to anneal to the universal smMIP backbone. smMIPs, synthetic oligonucleotides and primer sequences can be found in Supplementary File S3. In total, seven out of the ten smMIPs resulted in successful capture and amplification, resulting in the expected amplicon lengths of 186–204 bp (see Supplementary Figure S1). One of the key outcomes of this pilot experiment was the detection of by-products that did not contain the inserted target sequence (self-circularized probes). Given the ~100 bp length difference from the expected amplicons, this issue was easily identified and could be tackled by purification; although, if occurring in large amounts, it can deplete a significant amount of available smMIPs. Moving forward, due to the nature of individual target amplification, it was difficult to compare and evaluate the overall expected capture efficiency as a combined panel. We, however, confirmed that capture was possible based on Locksmith design and gained valuable insights into the capture process.

### 2.3. Capture Assessment of the Full 514-CpG smMIP Panel

Based on the successful capture of the pilot panel, we next aimed to employ the full panel in a single-reaction capture of all 514 age-associated CpGs. Ideally, capture optimization should be performed using the full smMIP panel to clarify the severity of off-target reads and self-circularization, as well as to improve the sequencing balance across targets. We aimed to identify the optimal capture condition for this 514-CpG smMIP panel in order to obtain methylation values for all target loci through uniform target capture. In total, 78 combinations of various capture parameters were tested (Supplementary File S2). smMIP hybridization success was evaluated by varying the smMIP concentration (0.25, 2.5 and 25 pM each), input DNA amount (10 and 100 ng) and incubation temperature (50, 53, 57 and 60 °C), while smMIP elongation success was tested by varying the dNTP concentration (5, 50 and 500 nM) and elongation time (2 h and 16 h).

**Figure 3:**
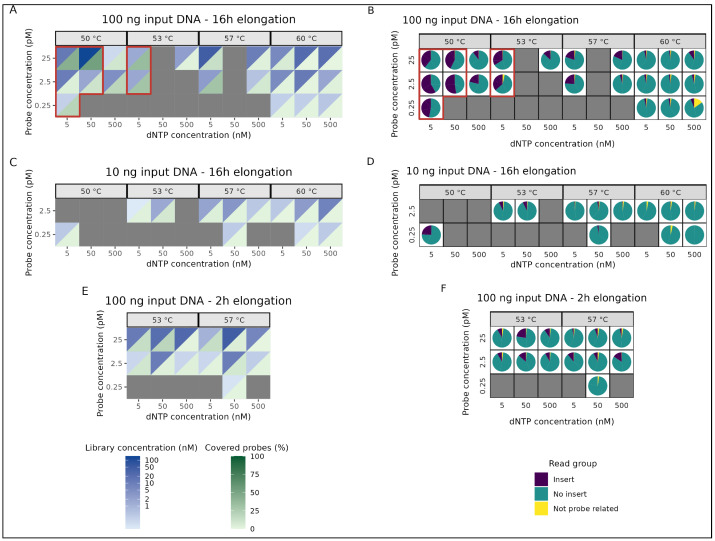
Performance of a total of 78 smMIP capture conditions. (**A**,**B**) 100 ng input DNA, 16 h elongation time. (**C**,**D**) 10 ng input DNA, 16 h elongation time. (**E**,**F**) 100 ng input DNA, 2 h elongation time. (**A**,**C**,**E**) Library concentrations of each capture condition are shown in blue, whereas the percentage of target probes that reach at least a 20× sequencing depth are shown in green. A darker shade of green indicates a higher target panel coverage. (**B**,**D**,**F**) Fractions of sequencing reads that either incorporate an insert, no insert or are not probe related (e.g. library preparation artifacts). Higher amounts of reads with incorporated inserts indicate more capture events and less self-circularization of smMIPs. Conditions for which the library concentration was too low for sequencing (<0.1 nM) are shown in grey. The red boxes mark the seven best-performing conditions.

Overall, 48 (62%) out of the 78 capture reactions resulted in sufficient output, meaning an amplicon library concentration of at least 0.1 nM, which was our minimum requirement for DNA sequencing. As expected, capture reactions with the highest input DNA (100 ng) and the longest elongation time (16 h) resulted in the highest target coverage. Here, 64% (23/36) of the reactions resulted in a sufficient library concentration (Figure 3A). Also, up to 58% reads of desired input length were acquired, indicating successful capture of an insert sequence (Figure 3B). Successful capture, by incorporating the target insert sequence, was strongly dependent on capture temperature and dNTP concentration. The amount of reads with inserts reached 58% and 36% for capture temperatures of 50 °C and 53 °C, respectively, whereas it was only 24% and 10% at 57 °C and 60 °C, respectively. In general, the extremely high dNTP concentration of 500 nM resulted in less to no incorporation of a target insert within smMIPs. Moreover, the smMIP amount seemed to have no impact on the capture quality but did result in higher DNA library quantities.

On the other hand, for the capture reactions using a lower input DNA amount (10 ng), only 50% (12/24) of the reactions resulted in a sufficient library concentration (Figure 3C). Regardless, the percentage of reads containing inserts only reached 24.2% for one condition (0.25 pM smMIPs, 5 nM dNTPS and a capture temperature of 50 °C), while it was 7.6% or lower for all other conditions (Figure 3D). Finally, the capture reactions with just 2 h of elongation time did result in sufficient library concentrations for smMIP concentrations of 2.5 and 25 pM (Figure 3E); however, similarly to the 10 ng input DNA reactions, the percentage of reads that successfully incorporated an insert was no more than 22.5% (25 pM smMIP, 50 nM dNTPs and a capture temperature of 53 °C) (Figure 3F). These percentages of reads that contain inserts for 10 ng input or a 2 h elongation time are all below 25%, whereas for the 100 ng input DNA with 16 h of elongation time, seven conditions are above this threshold, indicating that there are more reads originating from self-circularized probes.

To summarize, we identified the top seven smMIP capture conditions, resulting in at least 25% of insert-containing reads, which we decided to further analyze. These included reactions with 100 ng template DNA with 16 h elongation time at either a capture temperature of 50 °C with dNTP concentrations of 5 and 50 nM or a capture temperature of 53 °C with a dNTP concentration of 5 nM.

Beyond capture evaluation, we also aimed to evaluate how uniform and successful our coverage was for all of our 514 targets by analyzing all mapped reads. Overall, target coverage was highly variable across all 78 conditions, ranging from zero to 60,472 reads per target. The seven best-performing conditions reached a coverage of at least 20 reads for 4% up to 54% of all targets (Table 1). The observed coverage for all capture conditions can be seen in Supplementary File S2. In general, it can be noted that a higher smMIP concentration of 25 pM resulted in a higher read depth. Here, more smMIPs were available for capture events, increasing the chance for the probes to bind at their target location. Also, the lower capture temperatures of 50–53 °C over 57 and 60 °C resulted in more successful smMIP capture events by incorporation of an insert sequence, instead of forming self-circularized probes. Taking into account all conditions, 448 out of the 514 smMIPs resulted in one or more reads, while 66 smMIPs did not result in any capture event no matter which capture condition was used. This indicates an issue with probe design, since it was not possible to perform any capture reaction with these smMIPs.

**Figure 4:**
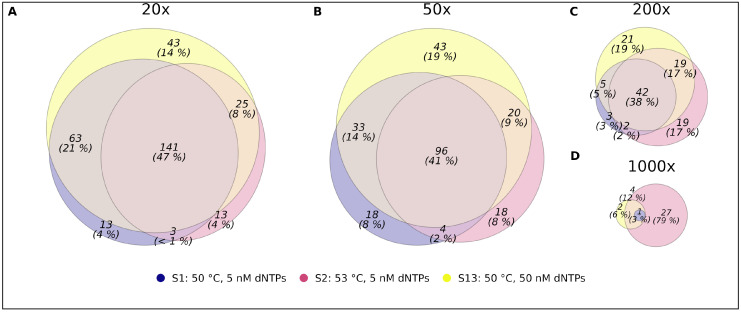
Venn diagram showing the overlap between the number of captured targets/CpGs across the top three best-performing smMIP capture conditions at read depths of (**A**) 20×, (**B**) 50×, (**C**) 200× and (**D**) 1000×.

Based on the results of the capture optimization experiment, we aimed to further analyze the seven best-performing conditions in more detail. The similarity of the read depth of the 514 smMIP targets across the top seven conditions shows that capture efficiency is mostly probe-specific (see Supplementary Figure S2). Similar coverages were obtained for each single smMIP regardless of capture condition. Additionally, the higher smMIP concentration of 25 pM resulted in the capture of more targets at higher sequencing depths, improving capture quantity. These three conditions with 25 pM of smMIPs also show an overlap of 141 captured targets (Figure 4). In the best-performing condition at 50 °C and 50 nM dNTPs, a total of 275 (54%) CpG targets reached a coverage of at least 20 reads, which has been shown to reach accurate beta values for NGS [30]. However, the uniformity of the capture efficiency was highly heterogeneous, with 334 of the 514 targets resulting in a 100-fold mean read-depth range (see Supplementary Figure S3).

Overall, the best capture conditions were reported at annealing temperatures of 50–53 °C, 5–50 nM dNTPs and 25 pM smMIPs. Although the highest target coverage was obtained at these conditions, only 275 of the 514 (54%) target CpGs reached a threshold of 20 reads in a single capture reaction. Therefore, the smMIP capture was not successful due to the following: 1—the presence of reads without target insert sequences; 2—no to low coverage, below 20×, for 213 (41%) of the 514 target CpGs in any reaction condition. The observation that the same probes reach higher read depths than others indicates that the capture efficiency is probe-dependent. From these results, it can be concluded that the capture efficiency is heterogeneous, indicating the need for probe balancing to increase the uniformity of target coverage.

### 2.4. Linking smMIP Design to Capture Efficiency

**Figure 5:**
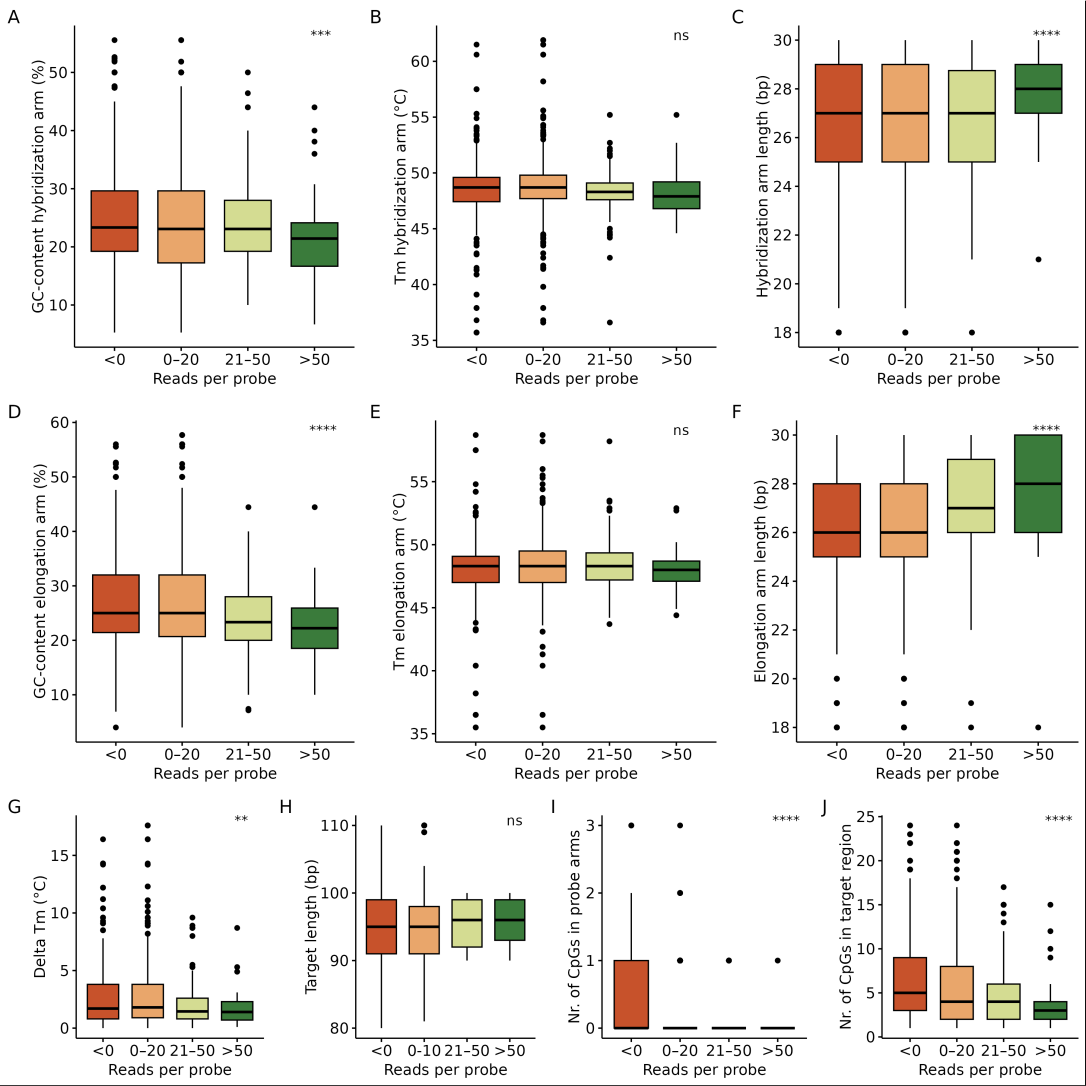
smMIP characteristics grouped by target coverage. (**A**) The GC-content, (**B**) melting temperature and (**C**) arm length of the hybridization arm. (**D**) The GC-content, (**E**) melting temperature and (**F**) arm length of the elongation arm. (**G**) Delta Tm of smMIP arms. (**H**) smMIP target length. (**I**) CpG number in probe arms. (**J**) CpG abundance in target sequence. ns: *p* > 0.05; ⁎: *p* ≤ 0.05; ⁎⁎: *p*≤ 0.01; ⁎⁎⁎: *p*≤ 0.001; ⁎⁎⁎⁎: *p*≤ 0.0001.

Next, with a goal to explain our findings and highlight possible strategies to further optimize probe design in future smMIP panels, we decided to correlate smMIP design parameters with the observed probe efficiency and off-target binding. To this end, the smMIPs were divided into groups based on the number of unique reads per capture condition (No reads: 0, 0–20 reads, 21–50 reads and more than 50 reads) (Figure 5). With the use of the unique molecular identifiers (UMIs), each unique read denotes a separate capture event. Therefore, duplicate reads can be used to correct for PCR-bias obtained during library preparation.

First, the smMIP design parameters were analyzed for their influence on the probe efficiency. A lower GC-content of both the hybridization and elongation arms of smMIPs were favored to reach a higher amount of capture events (*p*: $5.75\times 10^{-4}$, $1.08\times 10^{-5}$, respectively) (Figure 5A,D). Although the melting temperatures of the probe arms have no significant effect on the target coverage (Figure 5B,E), a 1 °C decrease in the difference between melting temperatures of the smMIP arms was positively correlated with increased read depths, as expected (*p*: $3.95\times 10^{-3}$) (Figure 5G). smMIPs that did not result in any reads had a delta Tm of 2.7 ± 2.7 °C between the probe arms, whereas smMIPs resulting in more than 50 reads had a delta Tm of 1.7 ± 1.5 °C. smMIPs with 2 bp longer probe arms resulted in higher read depths, likely due to the increased specificity, both for the hybridization probe arms (*p*: $9.02\times 10^{-7}$) and elongation probe arms (*p*: $3.19\times 10^{-9}$) (Figure 5C,F). smMIPs that did not result in any reads had arm lengths of 26 ± 3 bp, whereas smMIPs resulting in more than 50 reads had arm lengths of 28 ± 2 bp. Finally, the amounts of both the CpGs in smMIP arms as well as within the target region were negatively correlated with the acquired read depth (*p*: $1.49\times 10^{-16}$ and $1.92\times 10^{-7}$, respectively), indicating that CpG-dense regions were harder to cover (Figure 5I,J). Furthermore, the target length varying between 80 and 110 bp in our panel was not correlated with read depth as may be expected (Figure 5H). Overall, the observed correlations between target coverage and smMIP design parameters highlight the importance of optimizing the probe design in future panels. However, the strongest correlations were found to disfavor CpG-dense regions, in both the probe arms and target region, a finding that agrees with a previous study [41].

**Figure 6:**
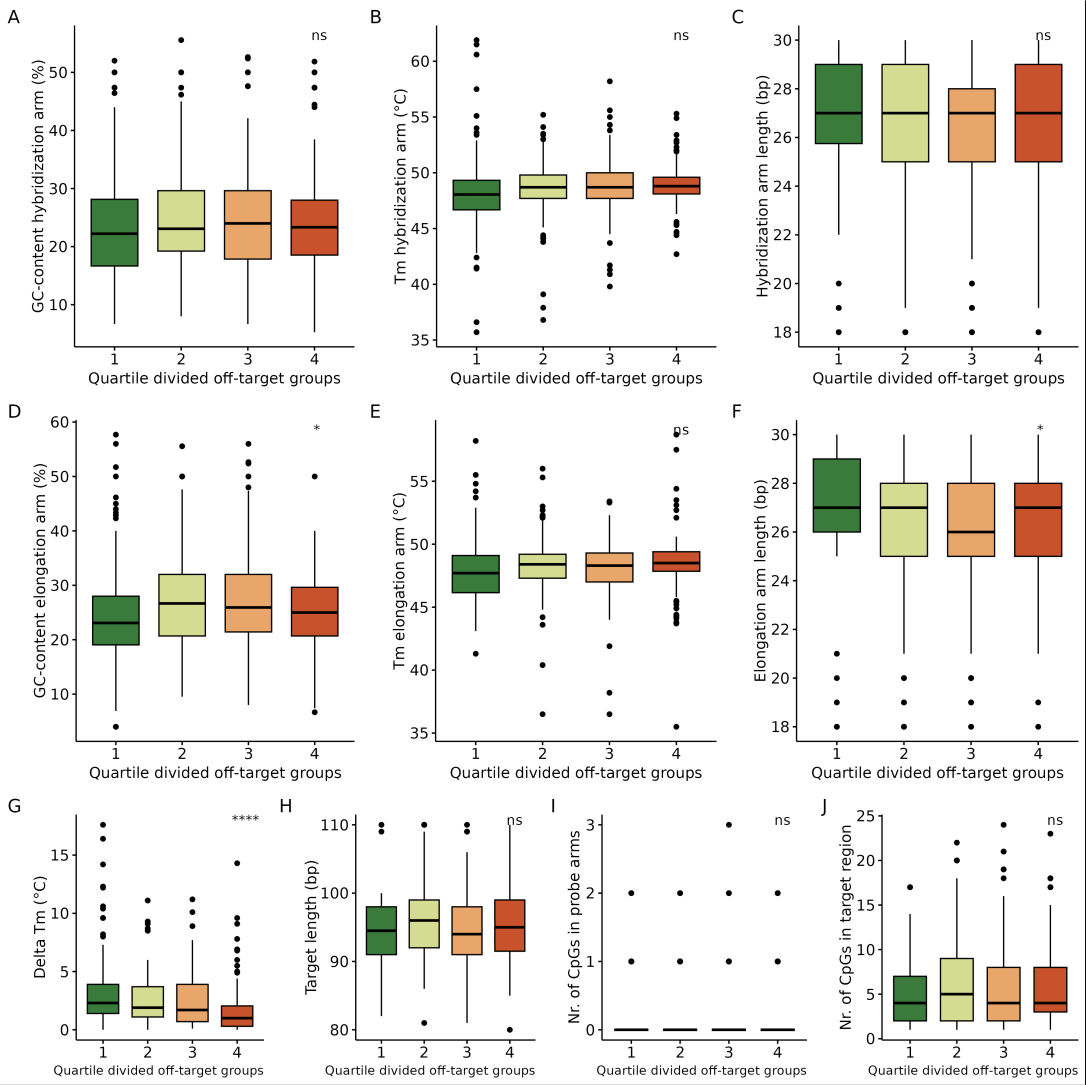
smMIP characteristics grouped by off-target abundance. The probes were ranked on the abundance of their probe arms in off-target reads. Four equal-sized groups were then created based on this ranking. Here, group 1 includes smMIPs that have the least contribution to off-target reads, whereas group 4 includes smMIPs with the most contribution. (**A**) The GC-content, (**B**) melting temperature and (**C**) arm length of the hybridization arm. (**D**) The GC-content, (**E**) melting temperature and (**F**) arm length of the elongation arm. (**G**) Delta Tm of smMIP arms. (**H**) smMIP target length. (**I**) CpG number in probe arms. (**J**) CpG abundance in target sequence. ns: *p* > 0.05; ⁎: *p* ≤ 0.05; ⁎⁎: *p* ≤ 0.01; ⁎⁎⁎: *p* ≤ 0.001; ⁎⁎⁎⁎: *p* ≤ 0.0001.

Second, to analyze whether the abundance of off-target reads could be explained by the smMIP design, data from the smMIP arms were aligned to the off-target reads. As a result, many reads were identified as being combinations of arm sequences from two different smMIPs, indicating the occurrence of chimeric capture processes. The smMIPs were then grouped into four equal-sized groups based on how often they resulted in off-target reads. Here, group 1 includes smMIPs that have the least contribution to off-target reads, whereas group 4 includes smMIPs with the most contribution (Figure 6). The most significant finding is the positive correlation of off-target reads with a lower delta Tm of the smMIP arms (*p* = $1.14\times 10^{-5}$) (Figure 6G). Since a lower delta Tm was previously found to correlate with a higher amount of target coverage (Figure 5G), balancing the delta Tm would be of major importance when designing an smMIP pool to maximize target coverage while minimizing off-target reads. Also, the GC-content plays a role in minimizing off-target reads. Although the GC-content of the hybridization arm was not significantly different between the four off-target groups, smMIPs with a lower GC-content in the elongation arm resulted in less off-target reads (*p* = $1.87\times 10^{-2}$) (Figure 6A,D). Additionally, while not true for the hybridization arm, a longer elongation arm length was significantly associated with lower off-target reads (*p* = $3.62\times 10^{-2}$) (Figure 6C,F), indicating that it stabilizes the capture. Finally, the melting temperature of the probe arms, target length and CpG density did not seem to influence the abundance of off-target reads (Figure 6B,E,H–J). Overall, the observed correlations between off-target read abundance and smMIP design parameters indicate the effect of the probe design on sequencing results. Here, especially, minimizing the delta Tm between the probe arms could reduce the number of off-target reads.

### 2.5. Comparison to the EPIC v2 Array

**Figure 7:**
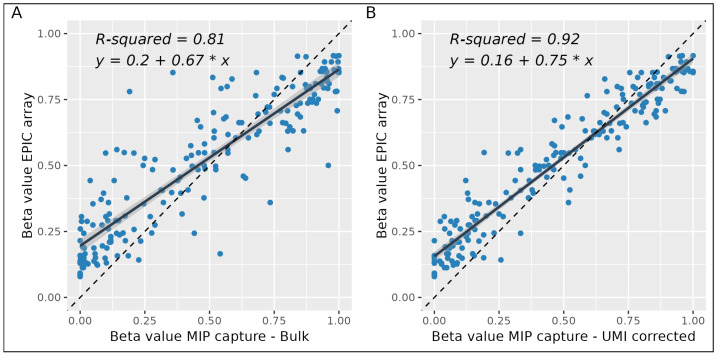
Comparison of observed DNA methylation levels between smMIP capture and EPIC array (**A**) without and (**B**) with UMI correction. Targets with at least 20 reads are shown (CpGs = 201).

As a final step, we aimed to explore whether the data obtained by our targeted methylation profiling method via smMIP capture are robust despite the low sequencing coverage. Therefore, we compared it with methylation data obtained through the current golden standard—Illumina’s EPIC v2 methylation beadchip array. For our analysis, we used 500 ng DNA and included the 275 targets with a read depth of at least 20× (Figure 7A). Overall, DNA methylation values showed a strong correlation between the two methods, as indicated by a R^2^ value of 0.81. This correlation only grew stronger to a value of 0.92 when only unique capture events were taken into account (after correction, using UMIs, for the introduced PCR bias) (Figure 7B). There seems to be a systematic underestimation of the beta values below 0.5 and an overestimation of the beta values above 0.5 for the smMIP capture method compared to the EPIC array approach. This systematic bias is most likely due to the disparity on the type of measurement between beadchip arrays and NGS approaches. Additionally, from the increase in Pearson’s correlation when considering unique capture events by the use of UMIs, PCR bias can be counteracted since multiple reads share the same UMI. Although this does decrease the read depth, DNA methylation values become more accurate. The effect of UMIs is highly evident on markers with a strong PCR bias, at which the use of UMIs can collapse over hundreds of reads back to a single capture event, e.g., the greatest outlier went from a beta value difference of 0.59 to 0.11 after the incorporation of UMIs. When more reads were obtained, DNA methylation levels showed a smaller spread around the linear regression, with the mean absolute error (MAE) decreasing from 0.058 at 20×–50× coverage to 0.046 above 50× coverage, thereby increasing accuracy (Figure S4A). Analogously, a wider spread of DNA methylation values was obtained at intermediate levels (0.2–0.8) with an MAE of 0.121, as opposed to 0.057 and 0.040 for the lower (<0.2) and higher methylation (>0.8) regions, respectively (Figure S4B). Although with UMI correction we reached an R^2^ value of 0.92, there were too many missing values to perform statistical modeling to determine the biological age of our sample using the 514-CpG Zhang model that we aimed for. Moving forward, marker selection or reduction prior to panel design and probe panel balancing could improve the number of markers that reach the coverage threshold.

## 3. Materials and Methods

### 3.1. smMIP Design by Locksmith

Locksmith is a python-based probe design tool for smMIPs, freely available at https://github.com/RoyBSimons/Locksmith. It is written as a 4-step Snakemake pipeline (v4.3.1) [42] using Python 3.9.5 and open-source available command-line tools. First, target sequences and their flanking regions are extracted by BEDTools v2.29.1 [43] from the human reference genome hg38 (NCBI RefSeq assambly GCF_000001405.40). Secondly, all possible combinations of probe arms are obtained and penalized based on the amount of included CpGs, number of common SNPs (>1%) in the arms by tabix 1.10.2 [44], possible hairpin formation by MFEprimer v3.2.6 [45] and delta Tm between the probe arms using the Tm_NN function from Biopython (1.79) [46]. Possible smMIP sequences are constructed by concatenating the hybridization arm sequence, unique molecular identifier (UMI) sequence of 9 degenerate nucleotides (NNNNNNNNN), backbone sequence and elongation arm sequence. We manually designed the backbone sequence to be compatible with the Illumina TruSeq adapter sequences, while minimizing basepair length. Thirdly, an smMIP pool is iterative chosen by randomly selecting smMIPs per target based on the penalty score. In each round, smMIPs with dimer-forming regions are reported by MFEprimer [45] and additionally penalized, thereby minimizing dimer formation in the final smMIP pool. Finally, a specificity check is performed using the command-line program nucleotide BLAST 2.14.0+ [47] to highlight unspecific smMIP sequences. The main input files are a target BED file, the human genome reference sequence and a configuration file, indicating the probe design parameters. Locksmith outputs the designed smMIP sequences and the probe characteristics in a summary CSV file (chosen_panel.csv).

To design a panel with highly similar smMIP characteristics, stringent design parameters were chosen—Target length: 90–100 bp; smMIP arm length: 25–30 bp; CG-percentage: 0–60%; and maximum Δ Tm of 5 °C and 10 °C between probe arms and between the full panel, respectively. Design parameters were loosened iteratively to accommodate the design for CG-dense regions. For the comparison with ppDesigner, the design parameters were also iteratively loosened. The parameters for both the ppDesigner and Locksmith panel design can be found in Supplementary File S3E. The Locksmith probe panel was ordered as a 10 pmol oPools™ Oligo pools by Integrative DNA Technologies (Coralville, IA, USA) (see Supplementary File S3B). Degenerate nucleotides were used for UMIs and for CpGs in the probe arms. The oligonucleotide pool was diluted with TE buffer pH 8.0 (Invitrogen, Waltham, MA, USA) to a 400 pM concentration per smMIP.

### 3.2. Whole Blood Sample

A whole blood sample was obtained from a healthy donor, following approval by the Medical Ethics Review Committee of the Erasmus MC University Medical Center Rotterdam. The blood sample was drawn in a 10 mL Blood Collection tube, BD Vacutainer^®^ with K2EDTA additive (Becton Dickinson, Franklin Lakes, NJ, USA), and stored at 4 °C for four weeks before being processed. Genomic DNA extraction was performed using the Paxgene^®^ Blood DNA kit (QIAGEN, Hilden, Germany) according to the manufacturer’s instructions. The genomic DNA extract was quantified in duplicate using the QuantiT™ PicoGreen™ dsDNA Assay kit and Varioskan LUX Multimode Microplate Reader (ThermoFisher Scientific, Waltham, MA, USA) according to the manufacturer’s recommendation. The extracted gDNA was stored at −20 °C before use. Consecutively, the gDNA sample was bisulfite-converted with the EZ DNA methylation kit (Zymo Research Corporation, Irvine, CA, USA) in separate reaction mixtures of 64 times for the 100 ng and 32 times for the 10 ng input DNA. Converted DNA samples were pooled post-conversion to create two ‘master’ samples, one for each DNA input, for analyzing various capture conditions to prevent introducing any bias due to the DNA sample or bisulfite-conversion. Quality control of the pooled conversion samples was performed by qBiCo [48], a novel multiplex qPCR assay previously developed by our group; specifically, the conversion efficiency was measured at 98% (See Supplementary Table S4). Converted DNA was used directly for smMIP capture.

### 3.3. smMIP Capture and Enrichment

The improved capture protocol designed by Biezuner et al. was followed with changes mentioned here [49]. To find the best-performing capture conditions, the pooled converted DNA sample was split into 36 portions of 100 ng and 18 portions of 10 ng. Each of these samples were tested at capture conditions varying in hybridization temperature (50, 53, 57 or 60 °C), smMIP pool concentration (0.25, 2.5 or 25 pM) and dNTP concentration (5 nM, 50 nM or 500 nM). Hybridization of the smMIP pools to the bisulfite-converted DNA of either 10 or 100 ng was performed in a 0.85× Epicentre Ampligase buffer (Immunosource, Schilde, Belgium) solution of 16 µL. The reaction mixtures were incubated in a thermal cycler at 98 °C for 3 min; followed by 85 °C for 30 min; 54, 57, 60 or 64 °C for 60 min; and 50, 53, 57 or 60 °C for 60 min, respectively. Gap filling was performed by the addition of a 9 µL pre-heated gap-fill mixture to each of the capture reactions, resulting in final concentrations of 5 nM, 50 nM or 500 nM dNTPs (New England Biolabs, Ipswich, MA, USA); 375 mM Betaine (Sigma-Aldrich, St. Louis, Missouri, USA); 1 mM NAD+ (New England Biolabs); 0.5× Ampligase buffer and amounts of 1.25 U Ampligase (Immunosource); and 5 U TaqNova Stoffel DNA polymerase (BLIRT, Gdansk, Poland). Mixtures were incubated at 50, 53, 57 or 60 °C for 16 h, respectively. Consecutively, a 1.8× AMPure XP (Beckman Coulter, Brea, CA, USA) bead cleanup was performed in order to remove enzymes. Hybridization, elongation and bead cleanup were performed directly after each other without any waiting step. Finally, digestion of linear smMIPs and non-circularized targets was performed by adding 8 U Exonuclease I (ThermoFisher Scientific) and 50 U Exonuclease III (ThermoFisher Scientific). Mixtures were incubated at 37 °C for 2 h and 40 min, followed by 80 °C denaturation for 20 min. Mixtures were stored at 4 °C before library amplification.

### 3.4. Library Preparation and Sequencing

Amplification of purified circularized targets with dual-matched indexed primers was performed to prepare single-step libraries for Illumina sequencing. This was achieved by a simultaneous amplification and adapter tagging step. The use of dual-indices minimizes cross-talk between samples, which is important for sensitive NGS applications, including for methylation analysis [50]. The primers bind on the backbone of all smMIPs, thereby universally amplifying the full panel. Specifically, circularized targets were amplified using 1× ZymoTaq premix with 1.25 µM of primers each in half volumes: 25 µL (primer sequences can be found in Supplementary File S3D). The PCR protocol included a 10-min denaturation step at 95 °C, followed by 38 cycles of 30 s denaturation at 95 °C, 30 s hybridization at 60 °C and 30 s elongation at 72 °C. A final extension was performed at 72 °C for 7 min. The PCR was optimized by testing various amounts of cycles (35, 38 and 40) and reaction volumes (25 and 50 µL) (see Supplementary Table S5). Following PCR, an additional purification step was performed with 1× AMPure beads, followed by two washes of freshly prepared 70% ethanol. Libraries were quantified by the KAPA library quantification kit (KAPA Biosystems, Wilmington, MA, USA) and pooled to a final concentration of 1 pM as the minimal input required for sequencing. The pooled library was sequenced using 150 bp paired-end sequencing with a NextSeq 2000 instrument (Illumina, Inc., San Diego, CA, USA) using the NextSeq P1 sequencing chip (Illumina, Inc.). A total 15% PhiX was spiked-in at sequencing to accommodate for lower amplicon sequence diversity due to bisulfite conversion. A total of 85 million sequencing reads was obtained ranging from 262 thousand to 4.03 million reads per capture condition with an overall sequencing quality of Q > 30 for 85.2% of reads.

### 3.5. 10-Probe Panel Capture Pilot

Before performing the capture with the full 514 target-CpG panel, a capture pilot was performed to optimize the capture reaction. Ten smMIPS from the full panel were manually chosen to represent probes of diverse characteristics including probes with melting temperatures at the highest variance and probe arms with degenerate nucleotides in CpG-dense regions. The sequences of the 10-probe panel can be found in Supplementary File S3A. A capture reaction was performed as described before, including hybridization, elongation with either 5 nM or 50 nM dNTPs, circularization and exonuclease treatment; however, no universal amplification was performed. In order to determine whether each of the ten targets was captured, the circularized smMIPs were amplified separately. Forward PCR primers were designed to anneal the target regions, while the reverse PCR primer was designed to anneal to the universal smMIP backbone, thereby only amplifying smMIPs with captured target sequences. A synthetic DNA fragment being targeted by smMIP 4 was ordered by IDT as a template for a positive control reaction. Target-specific primer sequences and the synthetic DNA target sequence for the 10-probe panel can be found in Supplementary File S3C. The universal reverse PCR primer sequence, V5_R_D701, can be found in Supplementary file S3D. PCR products were visualized on a 1% agarose gel electrophoresis after applying 300V for 1 h and 30 min using the PerfectBlue™ Horizontal Midi Gel System (PEQLab, Erlangen, Germany).

### 3.6. DNA Methylation Analysis by Lockpick

To perform DNA methylation analysis for each of the target sequences included in our panel, we constructed Lockpick, a Snakemake pipeline for smMIP-derived sequencing data analysis using Python 3.9., freely available at https://github.com/RoyBSimons/Lockpick. Demultiplexed FASTQ files together with the summary smMIP pool file obtained by Locksmith (chosen_panel.csv) and human reference genome hg38 (NCBI RefSeq assambly GCF_000001405.40) form the input for performing the analysis. First, adapters are cut off using cutadapt with the following flags to cut off the adapter sequences with default settings: “-a AGATCGGAAGAGCACACGTCTGAACTCCAGTCAC -A AGATCGGAAGAGCGTCGTGTAGGGAAAGAGTGT” [51]. This is followed by the removal of (oligo)nucleotides, which are leading or trailing the smMIP arm sequences, thereby trimming the amplicon sequences to the captured insert sequences flanked by the smMIP arms. Log files of this trimming step are recorded by the cutadapt info-file flag for the later performed off-target analysis. Additionally, the 9 bp unique molecular identifiers (UMIs) are extracted from read 1 to prepare the reads for mapping. Secondly, an amplicon reference file is created from the human reference genome and the summary smMIP pool file using BEDTools v2.29.1 [43]. For bismark to function on amplicon reference files, the sequences are padded with ‘NNNN’ at both ends. The reads are then mapped to the expected amplicon sequences as reference genome using Bismark with the non_directional flag: N = 1; D = 15; R = 2; score_min = L, 0, −0.2; and I = 100 [52,53]. Thirdly, for all CpGs in the target regions, the amount of methylated and unmethylated sites is reported by Bismark methylation extractor. Finally, the methylation values of the target CpGs only is returned as a tab-delimited Bismark Coverage report.

### 3.7. Genome-Wide DNA Methylation Analysis

To validate the DNA methylation values obtained by smMIP capture, 500 ng of gDNA was bisulfite converted and used as template DNA for genome-wide analysis using the Infinium MethylationEPIC v2.0 Kit (Illumina, Inc.), following manufacturer’s recommendations. Control normalization of DNA methylation (beta) values was performed with the preprocessIllumina function of the minfi 1.52.0 library [54] using the manifest file obtained from the IlluminaHumanMethylationEPICv2 1.0.0 library in R version 4.4.1.

### 3.8. Data Analysis

Statistical analysis was performed in R version 3.6.3. To find out the reason for our low-read mapping rate, an off-target analysis was performed. The reported smMIP-arms in each read recognized by cutadapt were used to group the reads into three groups. In the case that the insert length—the length between both arms—was below 5 bp, the read was regarded as having no insert. If no smMIP-arm sequence was found in the read, the read was reported as ‘not probe related’. These two qualifications divided the reads into three groups: (1) reads with insert sequences, (2) reads without an insert, denoting self-circularized probes and (3) non-identified origin products. In order to find which of the probe design parameters had an effect on the obtained target coverage, smMIPs were stratified into four groups: (1) no reads, (2) 1–20 reads, (3) 21–50 reads and (4) >50 reads. The significance of the effect that probe design parameters had on the reached coverage per probe was determined by applying ANOVA by the compare_means function from the ggpubr (0.6.0) library. In order to find which of the probe design parameters had an effect on the amount of off-target reads, smMIPs were stratified into four equally sized groups, based on how often the smMIP probe arms were found in off-target reads. Here, group 1 included smMIPs that have the least contribution to off-target reads, whereas group 4 included smMIPs with the most contribution. To determine the significance of the effect that probe design parameters had on the off-target reads originating from an smMIP, ANOVA was applied by the compare_means function from the ggpubr library. Figures included in the manuscript are generated with the ggplot2 (3.5.1), ggh4x (0.2.8), ggpubr, gtable (0.3.0) and gridExtra (2.3) libraries. Proportional Venn diagrams were generated with the eulerr (7.0.2) library. Pearson’s correlations regarding the obtained DNA methylation values from smMIP capture and beadchip array were calculated with the base R cor function. Finally, the linear model to correlate the DNA methylation values from both methods was calculated with the lm function from the base R stats library.

## 4. Discussion

In this study, we investigated smMIPs to develop a targeted approach for sensitive, medium-scale DNA methylation detection from low amounts of input DNA. To achieve this, we first created a novel smMIP panel design tool (Locksmith) to enable more specific, efficient and uniform capture. As a result, we were able to minimize the variance of the arm melting temperatures and minimize the amount of CpGs at annealing sites. Using 514 age-associated CpG sites as an example panel, we investigated various conditions to improve the capture, i.e., by varying capture duration, input DNA amount, annealing temperature, and dNTP and smMIP concentrations. Despite our thorough optimization efforts, various challenges remain to be solved before this approach can be applied to targeted epigenomic analysis of low-input DNA.

Firstly, the main hardship of successfully applying smMIPs to bisulfite-converted DNA is the amount of off-target reads that we obtained. At our best-performing condition, only 58% of the sequencing reads contained inserts that were mappable. Notably, this issue has not been reported when previously applying smMIPs for SNP detection, where the double hybridization arms resulting in higher specificity is actually the main strength rather than weakness compared to other targeted approaches [33,55]. When applying smMIPs to DNA methylation markers using bisulfite-converted DNA, the reason for the non-specific binding of the smMIP arms is the highly reduced complexity of the DNA template sequence after bisulfite conversion. Recently, similar low amounts of mapped reads (37 ± 12%) and on-target reads (1–22%) were found using MIPs for DNA methylation analysis [30]. Further improvements could still be made on the smMIP design of Locksmith to decrease the amount of off-target reads. Longer and lower GC-content of the elongation arm length are expected to result in a higher amount of on-target reads and, hence, a lower amount of off-target reads. Indeed, Boyle et al. noted that longer hybridization arms than elongation arms in their SNP-based smMIP panel resulted in a higher read count [56]. In our data, when combining the results from both correlations between smMIP parameters with read depth and off-target effects, longer elongation arms with lower GC-content were indeed favored. However, in CpG-dense regions, which are often of interest, designing smMIPs with such characteristics will be practically impossible.

Additionally, we obtained a lower amount of reads for CpG-dense target regions. A possible solution to this issue would be to be more flexible in the target length as an input parameter for Locksmith. A greater target length range increases the amount of probe arm possibilities, thereby giving more options to customize the probe panel. Increased target regions, however, will restrict applicability, as it provides challenges for analyzing short or fragmented DNA as typically confronted within liquid biopsies and forensic traces. At the same time, longer target lengths resulting in longer amplicon sizes will complicate library preparation and increase sequencing costs due to the need for longer sequencing reads. Also, we not only found high rates of off-target probe capture, but also of probe self-circularization (reads without inserts), a phenomenon mostly seen at higher incubation temperatures and dNTP concentrations. We hypothesize that the elevated amounts of dNTPs could have resulted in ‘crowding’ of the reaction mixture, thereby causing no or non-specific probe binding. This creates small to no-insert-containing and chimeric amplicons (comprising arms from two smMIPs). Similarly, at higher temperatures, solution dynamics are increased, so that smMIPs can partially or loosely bind at various genomic regions or stay in solution, giving the possibility for the DNA polymerase and ligase to circularize. Short off-target inserts were likely favored over long off-target inserts, as probe arms need to bind for shorter times before elongation and ligation.

Beyond improving capture based on bisulfite-converted DNA, another solution to the reduced complexity problem would be to employ a recently developed new conversion method based on Tet-assisted pyridine borane sequencing [57]. This conversion method retains the complexity of the genomic sequence in its majority as it solely converts methylated cytosines, as opposed to all non-methylated cytosines during bisulfite conversion. Although this Tet-assisted pyridine borane conversion has been applied in combination with various sample types and methods such as whole-genome sequencing [57,58,59], cell-free DNA sequencing [60] and long-read sequencing [61,62], successful implementation and validation by independent research groups is still lacking. During the initial stages of our study, we aimed to implement Tet-assisted pyridine borane conversion but were unable to successfully reproduce the conversion when using either commercially available hTET2 or in-house produced mTET1 enzymes, according to the protocol published by Liu et al. (see Supplementary Figure S5 and Table S1). Given that a commercially available kit for this conversion method is yet to be developed, its wider use is currently on hold. In addition, various other novel conversion methods have been proposed, such as five- and six-letter seq [63] and single-enzyme methylation sequencing (SEM-seq) [64]. Both of these methods keep the complexity of the four-letter genome as opposed to bisulfite or enzymatic conversion of most of the cytosines in the genome, decreasing the complexity for further downstream analysis. This retained complexity would make it possible to perform smMIP capture of CpGs with the same specificity and uniformity as shown in SNP detection.

Secondly, considering successful capture events in our data, the uniformity between targets is low, showing a heterogeneous coverage distribution, with reads varying greatly from zero to over two thousand reads per target. Especially, we failed to obtain any data for 13% of our targets (66 smMIPs). At the best condition, a maximum of 54% (275/514) of the targets reached a 20× coverage. This is similar to the three sequencing experiments involving MIP capture performed by Pospiech et al., with an initial 33% (53/161) of targets reaching this 20× coverage and 62% (100/161) and 24% (39/161) after probe panel optimization [30]. While we acknowledge the limitation that our presented method is only conducted on one healthy donor, we do not expect major differences in performance when samples from different donors are analyzed, since we see major differences in capture efficiency that depend on the capture conditions, which are independent of the DNA sequence. Probe capture efficiency could have been affected by a rare genetic variant in the donor’s DNA sequence or a stochastic effect of more than average DNA degradation at one specific target site, which could result in slight deviations on capture performance. Although there are various smMIP design parameters that indicated a greater likelihood for a sufficient capture, such as increased GC-content of the probe arms and reduced CpGs in both the probe arms and target sequence, it might not be possible for all targets to fit these criteria due to the significant constraints of the surrounding DNA sequence. Currently, it is common practice to perform empiric and iterative design of ‘difficult’ probes, since there is no sufficient probe capture efficiency predictor [65]. Additionally, probe panel balancing could improve target uniformity by adding low-efficiency probes in higher concentrations or by designing several probes per target (tiling), as used in other capture approaches (i.e., hybridization capture) [66,67]. Another solution to increase target uniformity would be the use of suppressor oligonucleotides—nucleotide sequences that block unavoidable off-target capture and reduce highly abundant on-target reads [26,30]. This, however, can become a time-consuming and costly process, which would need to be performed for each new target panel, decreasing overall user-friendliness. For less complex phenotypes than aging, it might be possible to take the complexity of the target region into account when translating them in practice, thereby increasing the chances of sufficient smMIP capture efficiency. It would also be possible to perform marker selection, while taking into account difficult to design regions, e.g., due to CpG density. A statistical model can then be retrained with a selection of markers. This accommodates more flexibility in the probe panel design, while taking into account the final outcome. Recently, Pospiech et al. compared the smMIP approach for a medium-scale CpG panel of 161 CpGs with two methods: hybridization capture and multiplex amplicon sequencing [30]. They showed that the multiplex amplicon sequencing method based on methylation Ion AmpliSeq chemistry (ThermoFisher Scientific) was more promising for successfully analyzing 100–150 CpGs from small amounts of input DNA (25 ng), without suffering from the coverage uniformity of high amounts of on- and off-target reads. While reaching the desired sequencing depth of 50 reads for 155 targets of their panel, six targets were not included due to design failures in difficult, repetitive parts of the genome [30]. The probe panel design for both the hybridization capture and multiplex amplicon sequencing approaches are proprietary; therefore, they are dependent on industrial partners for method development.

Thirdly, the DNA methylation values obtained with our smMIP approach might not be directly applicable in practice when using prediction models built with data obtained by other methods like methylation microarrays. This widely known method-to-method bias, which also exists for other methods, is intrinsic to the quantitative nature of DNA methylation analysis. As shown in our data, the employment of UMIs to correct and improve methylation detection accuracy might solve the problem, resulting in higher data correlation. However, the spread of methylation values obtained by the golden standard EPIC array was narrower than that of our sequencing approach, which was also noted by a previous comparison between whole-genome bisulfite sequencing and methylation microarray data [68]. We noted the same overestimation of low methylation levels (0–0.5) and underestimation of high methylation levels (0.5–1). This systematic difference originates from the raw fluorescence-based normalization needed for methylation beadchip array data. When using NGS, beta values around the limits of 0 and 1 can be more accurately measured due to the read-based beta value calculation. Although this systematic difference complicates the use of existing models trained on array data, it can be accounted for by data transformation [30]. Another important factor is the required resolution and reproducibility of the obtained methylation signals. For human CpG methylation, coverages of 250× to 1000× are recommended due to the frequently observed subtle methylation effects [69,70]. However, sequencing depths of only 15× in WGBS were sufficient to approximate microarray-generated methylation values [68]. Using only 15 reads as a threshold for determining DNA methylation levels only reaches a resolution of 6.7% methylation difference between samples, so it may be problematic for investigating smaller effect sizes. Additionally, for obtaining optimal DNA methylation values, UMIs need to be taken into account to correct for PCR bias requiring 15 reads to originate from unique capture events.

Obviously, the required resolution is highly tied to sensitivity, since it relies on both the probe capture efficiency and template DNA amount. In general, the library concentration and number of covered targets was low for capture conditions involving 10 ng template DNA, often resulting in insufficient DNA library concentrations for sequencing compared to the most successful capture conditions with 100 ng of template DNA. The 10 ng template sample covering the most target CpGs reached a 20× coverage threshold for only 40 targets (8%) as opposed to 275 targets (54%) for the best-performing 100 ng condition. Also, the highest percentage of reads that incorporated target inserts was 24% as opposed to 58% for 100 ng template DNA. These data show that both the capture quantity and quality decrease at a lower template amount. Ion Ampliseq, however, exhibits the lowest variation in DNA methylation levels at 25 ng of template DNA when targeting 155 CpGs [30]. Even lower template DNA amounts of 1–5 ng were reached through manual primer design, although only for interrogation of a maximum of 32 amplicons [71]. This suggests that a multiplex amplicon approach may be more suitable than smMIP capture for the targeted DNA methylation detection of 100–150 CpG sites.

## 5. Conclusions

In this study, we investigated single-molecule molecular inversion probes (smMIPs) for medium-scale targeted DNA methylation analysis using the example of a 514-CpG panel for age prediction. We developed a novel smMIP design tool (Locksmith), thoroughly optimized smMIP capture towards lower DNA amounts (10 ng) and developed a suitable data analysis tool (Lockpick). We found that the current method can interrogate up to 275 out of 514 (54%) target CpGs in the designed smMIP panel, considering 100 ng of template DNA and 20× sequencing coverage. smMIP-capture for DNA methylation analysis, in contrast to its previous use for SNP analysis, suffers from high rates of off-target reads, likely due to unwanted self-circularization and non-specific binding of smMIPs. With the low complexity of converted DNA, it is not recommended to perform an smMIP-capture-based approach on currently used bisulfite (or enzymatic)-converted DNA without investing substantial efforts in optimizing the probe design and capture processes. With the arrival of novel conversion methods that retain sequence complexity, such as TAPS and 5/6-letter-seq, it is worth further investigating smMIP capture for DNA methylation detection in the future.

## Figures and Tables

**Table 1:** Number of captured targets/CpGs at different sequencing depth levels for the seven best-performing capture conditions.

[Probes] (pM)	Temperature (°C)	[dNTP] (nM)	Read Depth				
			1000×	200×	50×	20×	1×
25	50	5	1 (0%)	54 (11%)	153 (30%)	226 (44%)	384 (75%)
		50	7 (1%)	87 (17%)	195 (38%)	275 (54%)	415 (81%)
	53	5	32 (6%)	83 (16%)	140 (27%)	184 (36%)	315 (61%)
2.5	50	5	0 (0%)	0 (0%)	2 (0%)	23 (4%)	183 (36%)
		50	1 (0%)	9 (2%)	28 (5%)	58 (11%)	160 (31%)
	53	5	42 (8%)	83 (16%)	123 (24%)	155 (30%)	248 (48%)
0.25	50	5	16 (3%)	47 (9%)	86 (17%)	107 (21%)	182 (35%)

## Data Availability

Locksmith, the smMIP panel design tool, is freely available at https://github.com/RoyBSimons/Locksmith. Lockpick, the targeted DNA methylation analysis tool suitable for sequencing data produced using Locksmith-generated smMIP panels, is also freely available at https://github.com/RoyBSimons/Lockpick, including the raw FASTQ files with the sequencing data of the three best-performing samples: S1 (50 °C annealing temperature and 5 nM dNTPs), S2 (53 °C annealing temperature and 5 nM dNTPs) and S13 (50 °C annealing temperature and 50 nM dNTPs).

## Supplementary Materials

The following supporting information can be downloaded at https://www.mdpi.com/article/10.3390/epigenomes9010008/s1, Supplementary File S1: S1_supplementary_file.pdf. Supplementary File S2: S2_supplementary_coverage_table.xlsx. Supplementary File S3: S3_supplementary_table_smMIP_design.xlsx.

## Abbreviations

The following abbreviations are used in this manuscript:

CpG	CG-dinucleotide
DMR	Differentially methylated region
NGS	Next-Generation Sequencing
smMIP	Single-molecule molecular inversion probe
SNP	Single nucleotide polymorphism
UMI	Unique molecular identifier
