# Supplementary material for: Investigating Single-Molecule Molecular Inversion Probes for Medium-Scale Targeted DNA Methylation Analysis
Source: Epigenomes. 2025 Mar 2;9(1):8. doi: 10.3390/epigenomes9010008 (PMC11941031; doi:10.3390/epigenomes9010008)
Supplement: Supplementary file 1 [file epigenomes-09-00008-s001.zip › S1_supplementary_file.pdf]

Supplementary Materials: Investigating Single-Molecule Molecular Inversion Probes for Medium-Scale Targeted DNA Methylation Analysis

Roy B. Simons [1], Hieab H. H. Adams [2,†], Manfred Kayser [1] and Athina Vidaki [1,‡,*]

S1. Supplementary information

S1.1. Single capture tests of 10-probe panel

While at the capture sample with 5 nM dNTPs targets 2, 3, 4, 5, 8 and 10 showed a band at the length of a captured target (Figure S1A), for the 50 nM dNTPs capture sample this is the case for targets 2, 3, 4, 5, 7 and 8 (Figure S1B). Additional bands are seen for all targets at a lower length of around 100 bp. These products are thought to be self-circularized probes. The hybridization arm of the probe binds, after which ligation of the 3′-end to the phosphorylated 5′-end occurs due to non-specific binding directly behind the 5′-end. This effect is not seen at the negative control PCR samples as no template DNA is available to aid ligation (Figure S1C).

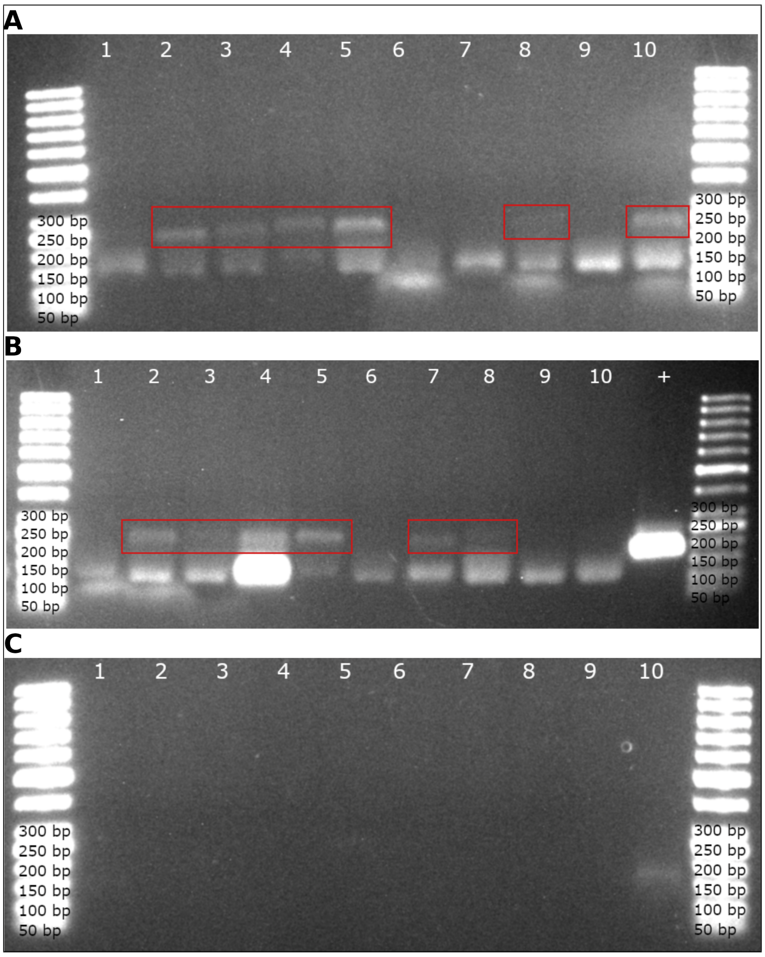

Figure S1. Target specific amplified products of a 10-probe capture reaction **A.** Amplified products of a capture at 5 nM dNTPs **B.** Amplified products of a capture at 50 nM dNTPs. **C.** Negative controls of target-specific PCRs containing no template DNA and 5 nM dNTPs. Top bands (In red boxes) indicate the expected products at expected lengths (186-204 bp). Bottom bands are unwanted products without target insert at probe lengths (98-109 bp).

S1.2. Probe capture efficiency

The probe capture efficiency of the full pool is condition dependent. However, probes that obtain high read depths in one condition also perform well in other conditions (See Figure S2). It is evident that the three conditions with a 25 pM probe concentration captured the most target CpGs, although at these conditions there is still a broad range of read depths.

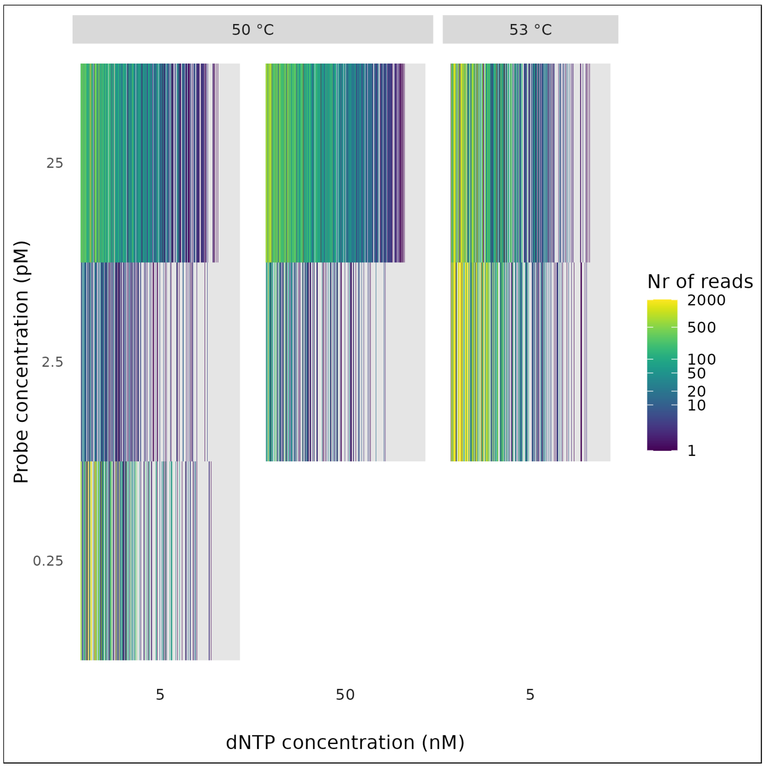

Figure S2. Visual representation showing the coverage of all 514 targets for the seven top performing MIP capture conditions. The targets are ordered on highest to lowest mean read depth.

The distribution of these read depths is visualized in Figure S3 averaged over the top three conditions. Here it can be seen that 192 of the 514 targets span a 10-fold range, whereas 334 span a 100-fold range.

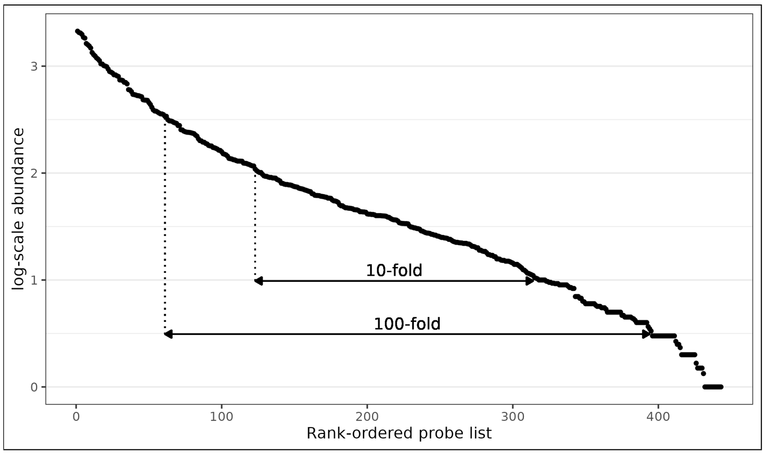

Figure S3. Uniformity of captured targets.

S1.3. Beta value spread

To delve into the origin of the disparity between the obtained beta values by the EPIC array and the smMIP capture, the distance between the smMIP obtained beta value and the

linear regression line is analyzed. The spread of the beta values obtained through smMIP capture show to decrease over an increase in coverage (S4A). The mean absolute error (MAE) is 0.058 at 20x-50x coverage to 0.046 above 50x coverage, indicating an increase in methylation percentage accuracy of ~1 percent point. Additionally, a higher spread is obtained at intermediate beta values over low (<0.2) or high (>0.8) beta values, with an MAE of 0.121, 0.057 and 0.040,respectively (S4B).

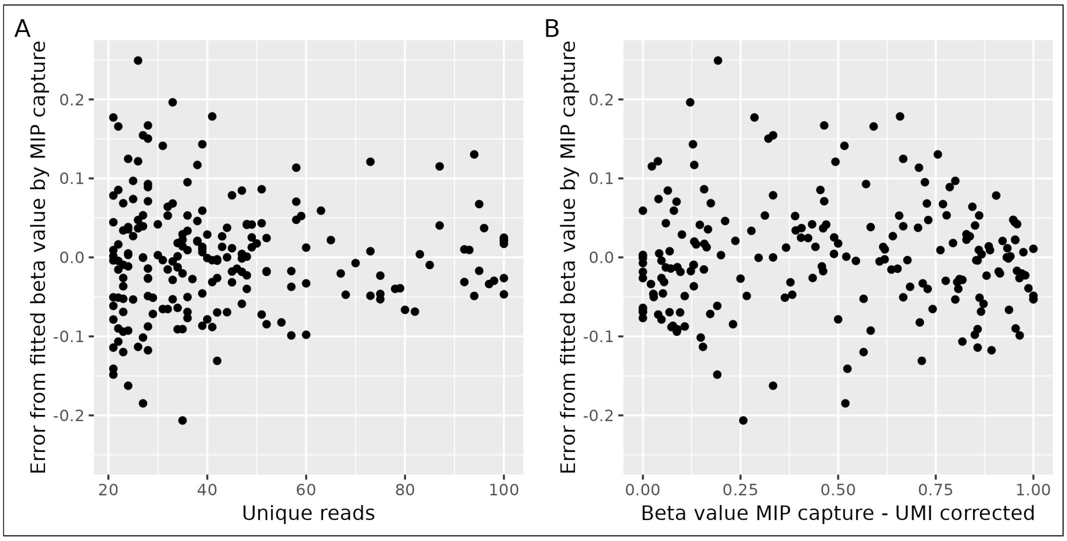

Figure S4. Beta value spread. **A.** The error from the fitted beta value decreases at higher read depths. **B.** The error from the fitted beta value increases at intermediate beta values.

S1.4. Tet-assisted pyridine borane conversion

As an alternative to bisulfite conversion, the novel Tet-assisted pyridine borane (TAP) conversion method was tested. TAP conversion only converts methylated cytosines to dihydroxy-uracils (5mC->DHU), leaving unmethylated cytosines unchanged, keeping the genome complexity for further downstream processing. mTET1 was produced according to the protocol [57].

In order to test the performance of TAP (Tet-Assisted Pyridine borane) conversion by qPCR, qTAP is designed. As 5mC is converted directly, an internal control, which can be added before conversion, is designed with high density 5mC regions. This is needed to have specific binding sites for primers and probes targeting either the converted or unconverted DNA. The designed internal control is a 400 bp long synthetic DNA fragment (See figure S5A). All CpG sites of the internal control were fully methylated by M.SssI (CpG methyltransferase; New England Biolabs, MA, USA). A methylation check was performed by restriction analysis with HpaII (New England Biolabs) as it is methylation specific. HpaII will not digest the DNA when the CpG is methylated (C▲CGG), but will when it is methylated, thereby confirming methylation. Primer sequences can be seen in Table S2. The Unconverted internal control sequence and its converted version for use in the qPCR standard can be seen in Table S3.

The performance of TAPS, was however, lower than expected (Table S1), with losses over 80% of the input DNA and conversion efficiencies of a maximum of 5.5% (Figure S5B). To verify it was not qTAP that malfunctioned, the Taqα1 assay was performed (Figure S5C) [57]. The partial digestion, indicated by the band around 400 bp, of the internal control indicates that there is some conversion, but most of the DNA is unconverted, thus digested.

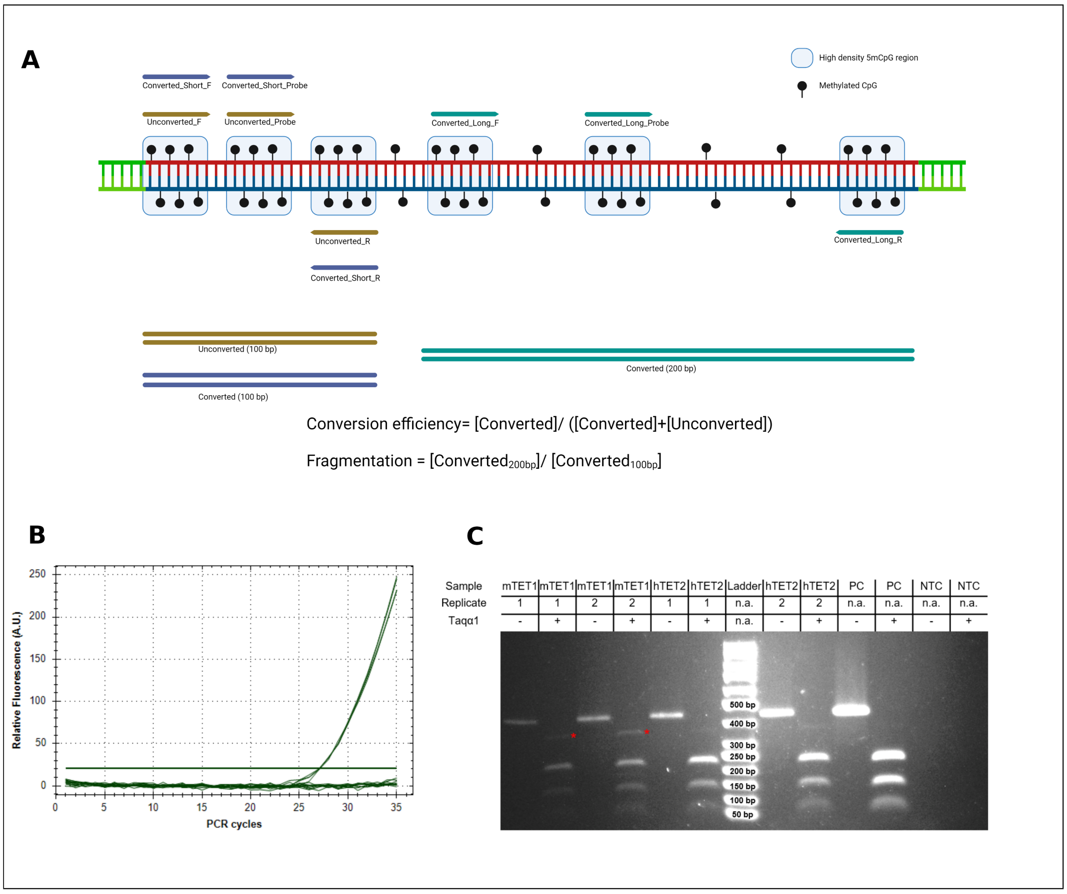

Figure S5. A. Schematic representation of the quality control qPCR assay qTAP. **B.** Amplification of converted DNA. **C.** Taqα1 assay showing partial digestion (Red asterisk) of internal control.

Table S1. Tet-assisted pyridine borane conversion performance for both in-house produced mTET1 and commercially available hTET2.

Sample	Replicate	Conversion efficiency	Recovery
mTET1	1	5.5 %	10.1 %
mTET1	2	5.1 %	10.8 %
hTET2	1	0.0 %	16.4 %
hTET2	2	0.0 %	12.1 %

Table S2. qTAP primer and probe sequences.

Name	Sequence	Tm °C
Unconverted_short_forward	CGTTCGAAACGATATTTATT	61.2
Unconverted_short_reverse	ATCGTAACGTATAACGATTA	60.5
Unconverted_short_probe_Cy5	5Cy5-GCGTCATCA-TAO-CGAACTCGAAT-3IAbRQSp	66.6
Converted_short_forward	CATTCAAAACAATATTTATTTACCCTCA	61.2
Converted_short_reverse	AATTGTAATGTATAATGATTATCCTGATTA	59.4
Converted_short_probe_HEX	5HEX-TGCATCATC-ZEN-ACAAACTCAAATACATGGCAT-3IABkFQ	66.1
Converted_long_forward	ACATATTCAATCCAGCAATACAGG	60.8
Converted_long_reverse	GGTGTATGATTGTATGGTGTACTG	60.1
Converted_long_probe_FAM	56FAM-TGCCTGACA-ZEN-ACAGTTGCACATCC-3IABkFQ	66.0
IC_Forward	CCTCATACACTTGGTTGCCA	61.2
IC_Reverse	AATACAGGCTGGTTAGTTGTC	60.6

Table S3. qTAP Internal control sequences.

Unconverted Internal Control (UIC)	CCAGGCCTCATACACTTGGTTGCCACGAGGCAGTCTCCGCGGTAAGTCCGTTCGAAACGATATTTATTTACCCTCGTCCTGCGTCATCACGAACTCGAATACGTGGCATTTTAATGTGGTAATCGGGATAATCGTTATACGTTACGATTTAGGCAGTGCATACTCTTCCATAAACGGGCTGTTAGTTATGGCGTCCGAGGATTCAAAAAGGTGAGCGAACGTATTCGATCCGGCGATACGGGCTTCAAAGCTGCCTGACGACGGTTGCGCGTCCAGAATCAAATCCTCCCAGGAATTGAGGCCGTCCGTTAATTTCCCTTGCATACATATTGCGTTATCTTGTCTGTATATCCGCTTACTTAGATAAGAGTGACATAGCTTCTTACCGGAGCGCCTCAGTACACCGTACGATCGTACGCCCCATGGACAACTAACCAGCCTGTATTGCTT
Converted Internal Control (CIC)	CCAGGCCTCATACACTTGGTTGCCACAAGGCAGTCTCCACAGTAAGTCCATTCAAAACAATATTTATTTACCCTCATCCTGCATCATCACAAACTCAAATACATGGCATTTTAATGTGGTAATCAGGATAATCATTATACATTACAATTTAGGCAGTGCATACTCTTCCATAAACAGGCTGTTAGTTATGGCATCCAAGGATTCAAAAAGGTGAGCAAACATATTCAATCCAGCAATACAGGCTTCAAAGCTGCCTGACAACAGTTGCACATCCAGAATCAAATCCTCCCAGGAATTGAGGCCATCCATTAATTTCCCTTGCATACATATTGCATTATCTTGTCTGTATATCCACTTACTTAGATAAGAGTGACATAGCTTCTTACCAGAGCACCTCAGTACACCATACAATCATACACCCCATGGACAACTAACCAGCCTGTATTGCTT

S1.5. Sodium Bisulfite conversion

Prior to MIP capture, the input DNA was bisulfite converted to be able to distinguish methylated from unmethylated cytosines. To ensure each of the MIP capture conditions were tested with the same input DNA, two master samples were created by pooling DNA from a multitude of bisulfite conversions of 100 ng and 10 ng input DNA. The performance of the conversion of these two pooled master samples can be seen in table S4.

Table S4. Bisulfite conversion performance of both the master samples with 100 and 10 ng input DNA.

Amount (ng)	Conversion Efficiency (%)	Intact DNA index	Recovery (%)
100	97.5	14.1	88.8
10	98.6	1.5	67.6

S1.6. PCR optimization

The PCR for library preparation was optimized by both varying the amount of PCR cycles and reaction volume. Both capture reactions starting with 100 ng and 10 ng gDNA template were tested. The library concentrations obtained from this optimization test can be seen in table S5. To reach a library concentration of 1 pM, it was decided to choose 38 cycles and 25 µl reaction volumes.

Table S5. PCR cycle optimization. Library concentrations are shown for 35, 38 and 40 PCR cycles for both 25 µl and 50 µl reaction volumes

**Input amount**	**100 ng**		**10 ng**	
**Reaction volume**	**50 µl**	**25 µl**	**50 µl**	**25 µl**
**35 cycles**	0.31	0.03	1.28	0.09
**38 cycles**	5.43	0.35	5.14	0.62
**40 cycles**	11.73	0.37	10.05	0.57
**PCR neg (40cycles)**	0.00	0.03	0.00	0.00
